# Impact of cooling method on the outcome of initial shockable or non-shockable out of hospital cardiac arrest patients receiving target temperature management: a nationwide multicentre cohort study

**DOI:** 10.1186/s13613-021-00953-y

**Published:** 2021-11-26

**Authors:** Makoto Watanabe, Tasuku Matsuyama, Hikaru Oe, Makoto Sasaki, Yuki Nakamura, Yuki Miyamoto, Nobunaga Okada, Tetsuhisa Kitamura, Bon Ohta

**Affiliations:** 1 Department of Emergency Medicine, Kyoto Prefectural University of Medicine, Kamigyo-ku, Kyoto, 602-8566 Japan; 2 Division of Environmental Medicine and Population Sciences, Department of Social and Environmental Medicine, Graduate School of Medicine, Osaka University, 2-2 Yamadaoka, Suita, Osaka 565-0871 Japan

**Keywords:** Target temperature management, Cooling method, TTM, Out-of-hospital cardiac arrest, Initial shockable rhythm, Initial non-shockable rhythm

## Abstract

**Background:**

Little is known about the effectiveness of surface cooling (SC) and endovascular cooling (EC) on the outcome of out-of-hospital cardiac arrest (OHCA) patients receiving target temperature management (TTM) according to their initial rhythm.

**Methods:**

We retrospectively analysed data from the Japanese Association for Acute Medicine Out‐of‐Hospital Cardiac Arrest registry, a multicentre, prospective nationwide database in Japan. For our analysis, OHCA patients aged ≥ 18 years who were treated with TTM between June 2014 and December 2017 were included. The primary outcome was 30-day survival with favourable neurological outcome defined as a Glasgow–Pittsburgh cerebral performance category score of 1 or 2. Cooling methods were divided into the following groups: SC (ice packs, fans, air blankets, and surface gel pads) and EC (endovascular catheters and any dialysis technique). We investigated the efficacy of the two categories of cooling methods in two different patient groups divided according to their initially documented rhythm at the scene (shockable or non-shockable) using multivariable logistic regression analysis and propensity score analysis with inverse probability weighting (IPW).

**Results:**

In the final analysis, 1082 patients were included. Of these, 513 (47.4%) had an initial shockable rhythm and 569 (52.6%) had an initial non-shockable rhythm. The proportion of patients with favourable neurological outcomes in SC and EC was 59.9% vs. 58.3% (264/441 vs. 42/72), and 11.8% (58/490) vs. 21.5% (17/79) in the initial shockable patients and the initial non-shockable patients, respectively. In the multivariable logistic regression analysis, differences between the two cooling methods were not observed among the initial shockable patients (adjusted odd ratio [AOR] 1.51, 95% CI 0.76–3.03), while EC was associated with better neurological outcome among the initial non-shockable patients (AOR 2.21, 95% CI 1.19–4.11). This association was constant in propensity score analysis with IPW (OR 1.40, 95% CI 0.83–2.36; OR 1.87, 95% CI 1.01–3.47 among the initial shockable and non-shockable patients, respectively).

**Conclusion:**

We suggested that the use of EC was associated with better neurological outcomes in OHCA patients with initial non-shockable rhythm, but not in those with initial shockable rhythm. A TTM implementation strategy based on initial rhythm may be important.

**Supplementary Information:**

The online version contains supplementary material available at 10.1186/s13613-021-00953-y.

## Background

Target temperature management (TTM) is a recommended treatment strategy to minimise the development of anoxic brain injury for out-of-hospital cardiac arrest (OHCA) patients [1, 2]. TTM can be induced and maintained with surface cooling (SC), such as ice packs, fans, cold air blankets, and SC pads or with endovascular cooling (EC), such as endovascular catheters and a blood temperature control device in dialysis techniques. While SC was used in two pivotal randomised studies that established TTM efficiency for OHCA patients [3, 4], EC was newly developed for more precise temperature management. Thus far, several studies have compared the impact of cooling methods on the outcome of OHCA patients with inconclusive results [5–10], suggesting the presence of uninvestigated confounding factors.

Recently, two large randomised controlled trials (RCT) that investigated the impact of the target temperature of TTM showed different conclusion between initial non-shockable patients and patients with all types of initial rhythms [11, 12], suggesting that the optimal target temperature strategy may be different for different initial rhythm. It is worth mentioning that there may be a potential interaction between cooling methods and initial rhythm on the outcome of OHCA patients. The known advantages of EC over SC are rapid induction and tighter temperature control during maintenance and rewarming phase [6, 9, 10], while the disadvantage is procedure-related complication [13]. These differences between cooling methods are largely derived from their different mechanism of heat exchange and their clinical significance may alter depend on patients’ physiological status. Since initial rhythm represents not only cardiac electrophysiological status but also various pre-arrest physiological status, such as no-flow time, obesity, rate of co-existing chronic condition, and age [14, 15]; initial rhythms may influence the impact of the cooling method on the outcome of OHCA patients. However, data on the effectiveness of the cooling method among patients with different initial rhythms are limited and, therefore, requires further investigation.

The aim of this study was to investigate the efficacy of SC and EC on the outcome of OHCA patients with initial shockable and non-shockable rhythm, using the database of the Japanese Association for Acute Medicine (JAAM)–OHCA Registry, a multicentre prospective registry.

## Methods

### Design, setting, and patient selection

We retrospectively analysed data from the JAAM–OHCA registry. The JAAM–OHCA registry is a multicentre, prospective, nationwide database that includes pre-hospital information, in-hospital information, and outcomes among OHCA patients transported to emergency departments in Japan. The registry started in June 2014 and is ongoing without setting the end date of the registry period. Currently, the registry includes 87 institutions: 66 of the included hospitals were university hospitals and/or critical care centres; the remaining 21 institutions were community hospitals providing emergency care at each community. The registry included all OHCA patients who were transported to the participating institutions and attempted resuscitation by emergency medical services (EMS). The registry excluded OHCA patients who were not resuscitated by a physician after hospital arrival, who were transported to a participating institution from another institution and who refused to participate in our registry, either personally or by family members. The protocol was approved by the institutional review board of each participating hospital. The registry was approved by the Ethics Committee of Kyoto University, and each hospital approved the JAAM–OHCA Registry protocol as necessary.

For our analysis, OHCA patients aged ≥ 18 years who were treated with TTM from June 2014 to December 2017 were included. Patients who were treated with extracorporeal membrane oxygenation (ECMO), who did not receive TTM and whose initially documented rhythm at the scene or applied cooling method were unknown were excluded.

### The EMS system in Japan

The EMS system in Japan has been described in detail previously [16]. Briefly, a crew of three emergency providers, including at least one emergency life-saving technician (ELST), are dispatched in each ambulance. ELST is a highly trained pre-hospital emergency care provider and is permitted to provide advanced life support, such as inserting intravenous lines or adjunct airways for patients with OHCA. Certified ELSTs after further training in hospitals are also allowed to administer intravenous epinephrine and perform tracheal intubation under online medical direction. Almost all OHCA patients cared for by EMS personnel are transported to hospitals and enrolled in the registry, because EMS personnel are not permitted termination of resuscitation (TOR) on the scene.

### Data collection

Prehospital data were collected by paramedics according to the international Utstein-style [17]. In-hospital data were collected by physicians or medical staff at each institution using a standardised format in an Internet-based system. The pre- and in-hospital information were integrated by the JAAM–OHCA registry committee, as previously described [18].

The following resuscitation-related data were used for this analysis: patient age, sex, cause of arrest (cardiac or not), presence of a bystander who witnessed the collapse of patient and who performed cardiopulmonary resuscitation (CPR), use of public accessed automated external defibrillator (AED), initially documented rhythm at the scene (shockable or non-shockable), prehospital epinephrine administration, prehospital advanced airway management, EMS response time (time from call to contact with a patient), use of ancillary cooling method (cold fluid for intravenous infusion or stomach cooling with nasogastric tube), performance of percutaneous coronary intervention (PCI) and whether the angioplasty was succeeded, targeted temperature during TTM (targeted at 32–34 °C as hypothermic TTM [H-TTM] or targeted at 35–36 °C as normothermic TTM [N-TTM]), cooling methods (SC or EC) applied for TTM implementation, and TTM induction time (time from initiation of cooling to achieving the target temperature). The TTM protocol was entirely entrusted to each physician or institution. The data collected as outcome measures were as follows: neurological outcome 30 days after cardiac arrest, survival 30 days after cardiac arrest, and completion of TTM. Neurological outcomes were evaluated using the Glasgow–Pittsburgh cerebral performance category (CPC) scale [19]: category 1, good cerebral performance; category 2, moderate cerebral disability; category 3, severe cerebral disability; category 4, coma or vegetative state; and category 5, death/brain death. TTM completion was defined as completion of each facility’s TTM protocol or discontinuation due to the recovery of consciousness.

### Outcome

In this study, the primary outcome was 30-day survival with favourable neurological outcome, defined as a CPC score of 1 or 2. The secondary outcomes were 30-day survival and TTM completion. In addition, we investigated outcome interactions between the cooling method and the initial rhythm to explore the heterogeneity of treatment effects.

### Statistical analysis

Cooling methods were divided into the following two groups: SC (ice packs, fans, air blankets, and surface gel pads) and EC (endovascular catheters and blood temperature control device in dialysis technique). If the cooling methods were duplicated, the TTM procedure category was defined as EC. We investigated the efficacy of two categories of cooling methods in two different patient groups divided according to their initially documented rhythm at the scene (shockable or non-shockable). Baseline patient characteristics and outcomes were evaluated using the Student’s *t* test or the Mann–Whitney *U* test for continuous variables after checking normality using the Kolmogorov–Smirnov test and Fisher’s exact test for categorical variables. To investigate the impact of the cooling method on each outcome, crude odds ratios (ORs) or adjusted odds ratios (AORs) and their 95% confidence intervals (CIs) were calculated by applying univariable and multivariable random effects logistic regression analyses with hospital treated as a random effect, by forced entry. Based on previous studies [20, 21], we adjusted for preliminary selected factors that were essential and considered to be associated with clinical outcomes, including age category (aged 18–64 years or aged ≥ 65 years), sex (men or women), cause of arrest (cardiac or non-cardiac aetiology), bystander witness (yes or no), bystander CPR status (yes or no), use of public-access AEDs (yes or no), prehospital adrenaline administration (yes or no), prehospital advanced airway management (yes or no), EMS response time (from call to contact with a patient), targeted temperature during TTM (H-TTM or N-TTM) and TTM induction time. Overdispersion was estimated by dividing the residual deviance by the degrees of freedom. To account for the nonrandomised selection of each cooling method, we also used propensity score methods to reduce the effects of confounding factors. The individual propensities for receipt of EC were estimated with the use of a multivariable logistic regression model that included the same covariates of basic and pre-hospital patients’ information as mentioned above for each of the overall cohort, shockable patients, and non-shockable patients (Additional file 1: Table S1). Associations between cooling methods and neurological outcomes were then estimated by univariable logistic regression analysis using inverse probability weighting (IPW). In addition, we calculated the OR in each subgroup and investigated the interaction effect to test for heterogeneity of the relative treatment effect across the particular patient characteristics of age category, presence of bystander witness, performance of PCI, and the targeted temperature. In a sensitivity analysis, we compared SC vs. intravascular device and SC vs. EC after excluding patients treated with both methods using the same model as the main analysis. As an ad-hoc analysis, we estimated the associations between cooling methods and 90-day outcomes using available data. All *P* values were two-sided, and the level of significance was set at 0.05. All statistical analyses were performed using R (The R Foundation for Statistical Computing, version 4.03, Saitama, Japan) and EZR (Saitama Medical Center, Jichi Medical University, version 1.54, Saitama, Japan), which is a graphical user interface for R [22].

## Results

**Fig. 1:**
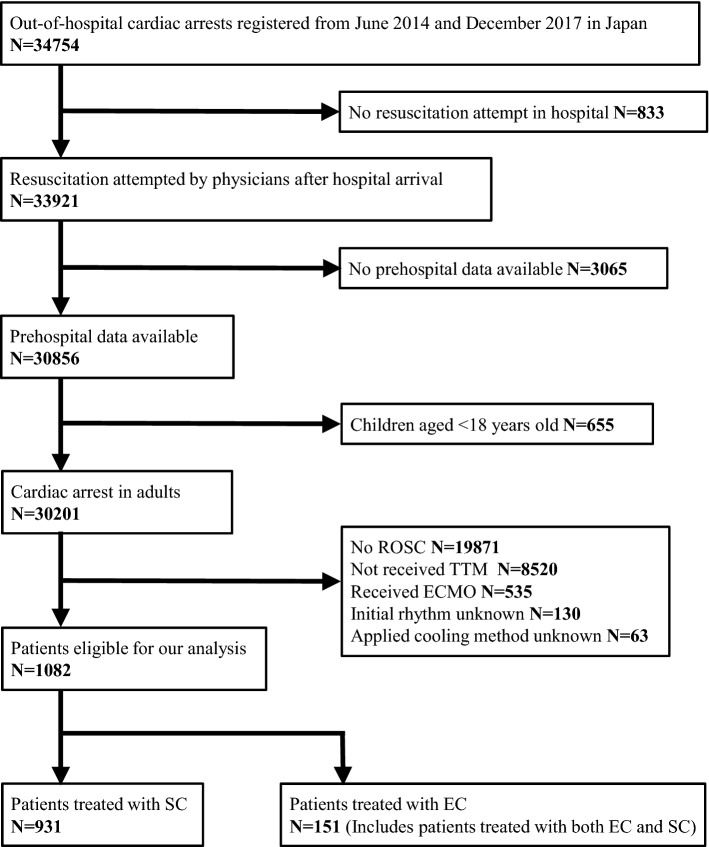
Study flow chart. *TTM* target temperature management, *ECMO* extracorporeal membrane oxygenation, *ROSC* return of spontaneous circulation, *SC* surface cooling, *EC* endovascular cooling

**Fig. 2:**
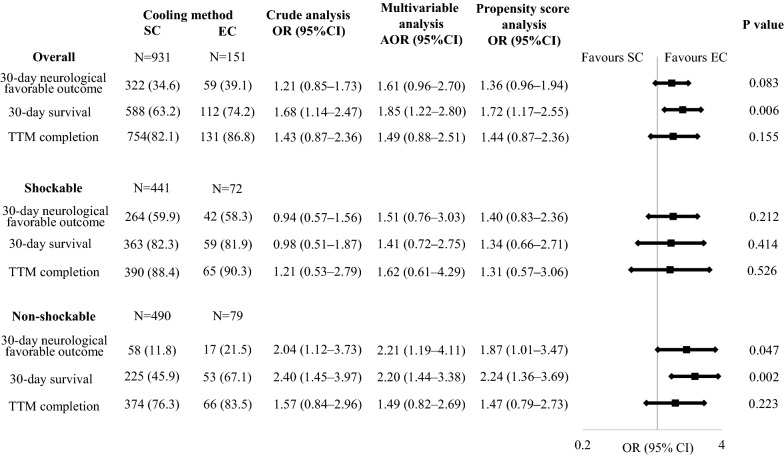
Outcome of the study population according to the cooling method. Values are expressed numbers (percentages) unless indicated otherwise. *TTM* target temperature management, *SC* surface cooling, *EC* endovascular cooling, *OR* odds ratio, *AOR* adjusted odds ratio, *CI* confidence interval. “EC” includes patients receiving both EC and SC methods. *Shown is the adjusted odds ratio from the multivariable random effects logistic regression analysis with hospital treated as a random effect, with stratification according to age, sex, cause of arrest, bystander witness, bystander CPR status, use of public-access AEDs, prehospital adrenaline administration, prehospital advanced airway management, EMS response time, target temperature and induction time of TTM. ^†^Shown is the odds ratio from the univariable logistic regression analysis with inverse probability weighting according to the propensity score

**Fig. 3:**
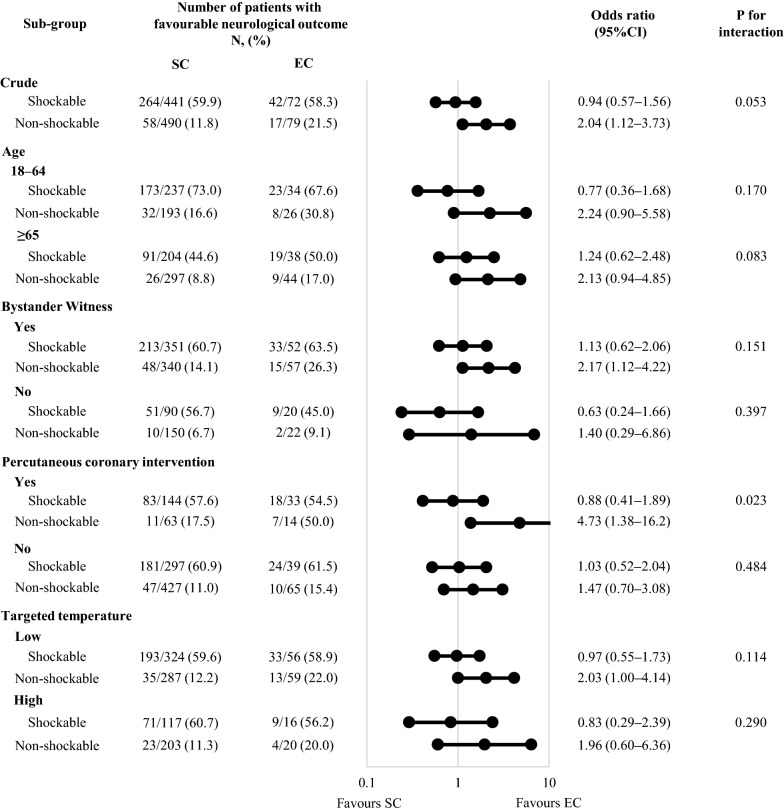
Interaction between initial rhythm and cooling method on the neurological favourable outcome of the patients. Values are expressed as numbers (percentages) unless indicated otherwise. *SC* surface cooling, *EC* endovascular cooling, *CI* confidence interval. “EC” includes patients receiving both EC and SC methods

A total of 34,754 OHCA patients were registered in the JAAM–OHCA registry between June 2014 and December 2017. After excluding 833 patients who were not resuscitated by physicians, 3065 patients whose prehospital data were not available, 655 patients aged under 18 years, 19,871 patients who did not get return of spontaneous circulation (ROSC), 8520 patients not receiving TTM, 535 patients who received ECMO, 130 patients whose initially documented rhythm was unknown, and 63 patients whose applied cooling method was unknown, 1082 patients were eligible for our final analysis (Fig. 1). Of the 1082 patients, 513 (47.4%) had an initial shockable rhythm and 569 (52.6%) had an initial non-shockable rhythm. The number of the patients treated with EC were, 151 (14.0%), 72 (14.0%), and 79 (13.9%) in the overall cohort, shockable rhythm, and non-shockable rhythm, respectively. Table 1 shows the baseline characteristics of the study population according to the cooling method. In the Kolmogorov–Smirnov test, distributions of the numerical variables were not normal. Baseline patient characteristics were similar in both cooling method groups, while patients treated with EC were more likely to receive prehospital epinephrine, PCI, and H-TTM. Standardised mean differences in baseline characteristics of the study population, defined as covariates for propensity score analysis, according to the cooling method before and after adjustment for IPW are shown in Additional file 1: Table S1. Regarding the primary outcome, the proportion of patients with favourable neurological outcomes treated with SC and EC was 34.6% (322/931) vs. 39.1% (59/151) in the overall cohort, 59.9% (264/441) vs. 58.3% (42/72) among initial shockable patients, and 11.8% (58/490) vs. 21.5% (17/79) among initial non-shockable patients (Fig. 2). In the multivariable logistic regression analysis, no difference was observed between the two cooling methods in the overall cohort (OR 1.61, 95% CI 0.96–2.70) and among the initial shockable patients (OR 1.51, 95% CI 0.76–3.03), while EC was associated with better neurological outcomes among the initial non-shockable patients (OR 2.21, 95% CI 1.19–4.11). The estimate of dispersion was 1.029, 1.174, and 0.833 in the logistic regression model for 30-day neurological favourable outcome, 30-day survival, and completion of TTM, respectively. In the propensity score analysis, no difference was observed between the two cooling methods in the overall cohort (OR 1.36, 95% CI 0.96–1.94) and among the initial shockable patients (OR 1.40, 95% CI 0.83–2.36), while EC was associated with better neurological outcomes among the initial non-shockable patients (OR 1.87, 95% CI 1.01–3.47). In addition, the impact of SC and EC on the outcome of the patients showed, though not significant, heterogeneity in different initial rhythms (*P* for interaction = 0.053), and the heterogeneity was significant among those patients who received PCI (*P* for interaction = 0.023) (Fig. 3). In the sensitivity analysis, we found that among patients treated with EC, 27 of 151 (17.9%) were simultaneously treated with SC and 37 of 151 (24.5%) were treated with dialysis techniques with a blood temperature control device. Our data showed that the result was consistent after excluding these patients (Table 2).

**Table 1:** Baseline characteristics of the study population according to the cooling method

	All patients	Missing	Overall		*P* values*	Shockable		*P* values*	Non-shockable		*P* values*
			SC	EC		SC	EC		SC	EC	
	*n* = 1082		*n* = 931	*n* = 151		*n* = 441	*n* = 72		*n* = 490	*n* = 79	
Basic information											
Men	801 (74.0)	0 (0.0)	692 (74.3)	109 (72.2)	0.617	356 (80.7)	56 (77.8)	0.527	336 (68.6)	53 (67.1)	1
Age, y, median (IQR)	66 (53–75)	0 (0.0)	65 (53–94)	67 (55–89)	0.288	63 (51–72)	66 (53–73)	0.192	68 (56–78)	59 (56–77)	0.742
Age category					0.158			0.312			0.319
Aged 18–64 years	490 (45.3)		430 (46.2)	60 (39.7)		237 (53.7)	34 (47.2)		193 (39.4)	26 (32.9)	
Aged ≥ 65 years	592 (54.7)		501 (53.8)	91 (60.3)		204 (46.3)	38 (52.8)		297 (60.6)	53 (67.1)	
Cardiac cause of arrest	777 (71.8)	0 (0.0)	664 (71.3)	113 (74.8)	0.435	420 (95.2)	69 (95.8)	1	244 (49.8)	44 (55.7)	0.602
Prehospital information		0 (0.0)									
Bystander witness	800 (73.9)	0 (0.0)	698 (74.2)	109 (72.2)	0.618	351 (79.6)	52 (72.2)	0.165	340 (69.4)	57 (72.2)	0.335
Bystander CPR	544 (50.3)	0 (0.0)	466 (50.1)	78 (51.7)	0.726	246 (55.8)	40 (55.6)	1	220 (44.9)	38 (48.1)	0.739
Use of public-access AEDs	82 (7.6)	0 (0.0)	70 (7.5)	12 (7.9)	0.868	41 (9.3)	6 (8.3)	1	29 (5.9)	6 (7.6)	0.532
Initially documented rhythm at the scene		0 (0.0)			1						
Shockable rhythm	513 (47.5)		441 (47.4)	72 (47.7)							
Ventricular fibrillation	505 (46.8)		433 (46.5)	72 (47.7)		433 (98.2)	72 (100)		N/A	N/A	
Pulseless ventricular tachycardia	8 (0.7)		8 (0.9)	0 (0.0)		8 (1.5)	0 (0.0)		N/A	N/A	
Non-shockable rhythm	569 (52.5)		490 (52.6)	79 (52.3)							
Pulseless electric activity	324 (29.9)		281 (30.2)	43 (28.5)		N/A	N/A		281 (57.3)	43 (54.4)	
Asystole	245 (22.6)		209 (22.4)	36 (23.8)		N/A	N/A		209 (42.7)	36 (45.6)	
Epinephrine	339 (31.3)	0 (0.0)	275 (29.5)	64 (42.4)	0.002	97 (22.0)	30 (41.7)	< 0.001	178 (36.3)	34 (43.0)	0.261
Advanced airway management	950 (87.8)	0 (0.0)	822 (88.3)	128 (84.8)	0.228	387 (87.8)	64 (88.9)	1	435 (88.8)	64 (81.0)	0.064
Call to EMS contact with a patient, min, median (IQR)	8 (6–9)	0 (0.0)	8 (6–10)	8 (7–9)	0.887	7 (6–9)	7 (6–8)	0.695	8 (7–10)	8 (7–10)	0.832
In-hospital information											
Use of ancillary cooling methods	417 (38.5)	0 (0.0)	349 (37.5)	68 (45.0)	0.087	189 (42.9)	38 (52.8)	0.126	160 (32.7)	30 (38.0)	0.369
Cold intravenous fluid	369 (34.1)		306 (32.9)	63 (41.7)	0.04	164 (37.2)	36 (50.0)	0.05	142 (29.0)	27 (34.2)	0.355
Stomach cooling with nasogastric tube	125 (11.6)		111 (11.9)	14 (9.3)	0.411	73 (16.6)	8 (11.1)	0.297	38 (7.8)	6 (7.6)	1
Percutaneous coronary intervention	254 (23.5)	0 (0.0)	207 (22.2)	47 (31.1)	0.022	144 (32.7)	33 (45.8)	0.033	63 (12.9)	14 (17.7)	0.286
Success of percutaneous coronary intervention^a,b^	231 (90.9)	9 (3.5)	189 (95.5)	42 (89.4)	0.153	134 (96.4)	29 (87.9)	0.07	55 (93.2)	13 (92.9)	1
Targeted at H-TTM	726 (67.1)	0 (0.0)	611 (65.6)	115 (76.2)	0.012	324 (73.5)	56 (77.8)	0.472	287 (58.6)	59 (74.7)	0.006
TTM induction time, min, median (IQR)^a^	150 (63–300)	42 (3.9)	150 (70–300)	120 (60–243)	0.03	180 (90–355)	150 (64–297)	0.05	129 (60–246)	99 (60–188)	0.295

Values are expressed numbers (percentages) unless indicated otherwise

*IQR* interquartile range, *CPR* cardiopulmonary resuscitation, *AED* automated external defibrillator, *EMS* emergency medical service, *TTM* target temperature management, *H-TTM* target temperature of 32, 33, 34 °C, *SC* surface cooling, *EC* endovascular cooling. “EC: *n* = 151/72/79” includes patients receiving both EC and SC methods

^*^Comparisons between the 2 groups were evaluated with Mann–Whitney *U* test for numeric variables and Fisher’s exact test for categorical variables

^a^Calculated for patients for whom data was available

^b^Calculated for patients who were performed PCI

**Table 2:** Sensitivity analysis comparing SC vs. subgroups of EC for the 30-day neurological favourable outcome

	All patients	Cooling method		Crude analysis	Multivariable analysis	Propensity score analysis	*P* value
		SC	EC	OR (95% CI)	AOR (95% CI)^a^	OR (95% CI)^b^	
SC vs. EC as endovascular device and excluding dialysis cooling							
Overall	378/1060 (35.7)	330/946 (34.9)	48/114 (42.1)	1.36 (0.91–2.02)	2.17 (1.31–3.60)	1.75 (1.18–2.59)	0.005
Shockable	304/504 (60.3)	270/452 (59.7)	34/52 (65.4)	1.27 (0.70–2.32)	1.89 (0.87–4.10)	1.75 (0.93–3.29)	0.081
Non-shockable	74/556 (13.3)	60/494 (12.1)	14/62 (22.6)	2.11 (1.10–4.06)	2.91 (1.43–5.91)	2.31 (1.20–4.43)	0.012
SC vs. EC after excluding patients treated with both SC and EC method							
Overall	369/1055 (35.0)	322/931 (34.6)	47/124 (37.9)	1.15 (0.78–1.70)	1.56 (0.87–2.80)	1.30 (0.88–1.91)	0.184
Shockable	296/495 (59.8)	264/441 (59.9)	32/54 (59.3)	0.98 (0.55–1.73)	1.45 (0.63–3.33)	1.34 (0.75–2.42)	0.325
Non-shockable	73/560 (13.0)	58/490 (11.8)	15/70 (21.4)	2.03 (1.08–3.83)	2.25 (1.18–4.32)	2.19 (1.17–4.12)	0.014

Values are expressed numbers (percentages) unless indicated otherwise

*TTM* target temperature management, *SC* surface cooling, *EC* endovascular cooling, *OR* odds ratio, *AOR* adjusted odds ratio, *CI* confidence interval

^a^Shown is the adjusted odds ratio from the multivariable random effects logistic regression analysis with hospital treated as a random effect, with stratification according to age, sex, cause of arrest, bystander witness, bystander CPR status, use of public-access AEDs, prehospital adrenaline administration, prehospital advanced airway management, EMS response time, target temperature, TTM induction time

^b^Shown is the odds ratio from the univariable logistic regression analysis with inverse probability weighting according to the propensity score

## Discussion

In this study, we assessed the impact of cooling methods at different initial rhythms on the outcome of 1082 OHCA patients, using the database of the JAAM–OHCA registry which included patients from 87 institutions in Japan. The main results of this study are summarised as follows: first, EC was associated with significantly better 30-day neurological outcomes compared with SC in patients with an initial non-shockable rhythm. Second, this difference was no longer apparent in patients with an initial shockable rhythm. Third, this association was constant in terms of 30-day survival of the patients. Our results confirmed the results of previous trials including three RCTs, [7–9] and furthermore, our study first focused on the heterogeneity of the effectiveness of the cooling method between patients with different initial rhythms and suggested that the optimal cooling method for TTM after OHCA may differ depending on the initial rhythm.

Initial non-shockable rhythm is known to be associated with longer no-flow time, which means longer exposure to global ischaemia [14]. Patients with an initial non-shockable rhythm are also associated with older age and a higher rate of co-existing chronic conditions that predispose to deterioration in underlying conditions and depletion of physiologic reserves [15], can be more susceptible to ischaemic injury. As ischaemic injury progresses, patients are imminently threatened with reperfusion injury [23, 24]. Therefore, the advantages of rapid and tighter temperature control of EC may be more prominent in patients with non-shockable rhythm.

Our patient demographics showed several differences from previous studies and may affect the outcome. First, the percentages of patients who presented with shockable rhythm (47% vs. 51%) and those treated with H-TTM (67% vs. 73%) were slightly decreased compared to previous report from a large international registry, possibly because of the secular change in the implementation of TTM [25]. Second, there is a very low proportion (31.3%) of epinephrine use. One possible explanation is that as pre-hospital management of OHCA patients largely depends on online direction and TOR is not allowed for EMS personnel, ‘scoop and run’ may be the most selected strategy when the patients seem to have a very low chance of survival or benefit more from early intervention by physicians. Third, only 1082 out of the 30,201 patients of attempted resuscitation at the hospital (3.6%) met our inclusion criteria. In our registry, almost all OHCA patients who were treated by EMS personnel were transported to a hospital, while field TOR was over 60% of all OHCA patients in other areas [26, 27]. Only one-third of the patients got ROSC after arrival at the hospital. Therefore, the patient demographics at hospital arrival may be more severe than in other countries, and thus, only a small proportion who got ROSC and were judged to tolerate active temperature control were included in our study.

The classification used by the authors, as well as previous trials, regarding the different TTM methods can introduce biases. Several trials with different classifications of EC, such as EC using intravascular devices [28], and EC using intravascular devices or dialysis techniques [29], showed superiority of EC compared to SC, and this result is consistent with our result. On the other hand, another meta-analysis comparing more recent SC devices, such as Arctic Sun®, with EC showed no difference between the two groups [30]. These results suggest a potential difference in effectiveness between newer SC devices and conventional SC methods, such as ice packs or fans. Exclusion of ECMO is another concern. The characteristics of patients treated with ECMO are different from those of other OHCA patients because of the limited indications [31], and the reported outcomes of these patients vary widely [32, 33]. However, most studies included in a meta-analysis report neither mentioned about the inclusion of ECMO patients nor provided data on the actual number of patients treated with ECMO [30]. Therefore, the impact of inclusion of ECMO patients on the results of these studies is immeasurable. As such, our results cannot preclude whether differences exist between endovascular cooling and more powerful recent SC methods such as advanced external devices or whether differences could be obtained with ECMO.

It is also worth mentioning that the number of surviving patients is higher compared to that observed in an international registry [25]. In Japan, withdrawal from treatment such as termination of mechanical ventilation is not generally accepted [34]. Therefore, the survival rate of patients with severe neurological injury without other organ failure, who would be withdrawn from life-prolonging treatment in other countries, will be higher. Another possible explanation is that we excluded patients treated with ECMO. Since this group of patients shows different outcomes from the usual OHCA patients [35], this exclusion might have increased the patients’ overall survival.

Despite several risks of biases, our study has some future implications. Considering the results of recent trials that focused on initial non-shockable patients that showed superiority of H-TTM [11, 36], and an RCT that include all types of initial rhythms showed no difference between N-TTM and H-TTM [12], the optimal TTM strategy may be different for different initial rhythm. As our data also suggest the heterogeneity of effectiveness of SC and EC between different initial rhythms, there may be certain patient groups who can benefit more from intensive TTM strategy. However, intensive TTM requires more logistics and costs and is not available everywhere. Therefore, our results underscore the need for further studies to identify a subset of patients who need intensive treatment strategies.

Our study has several limitations. First, as discussed above, the classification of cooling methods can introduce biases. Although we performed several sensitivity analyses in accordance with different EC classifications showing consistent results, we do not have the detailed data of the cooling method used in the SC group, and we excluded patients treated with ECMO. Therefore, whether this difference can be observed when SC devices are limited to the recent, more precise temperature control devices and when the patients treated with ECMO are included is unknown. Second, due to the heterogeneity of cardiac arrest status in our patients (among our study patients, 26.1% were unwitnessed, 49.7% were without bystander CPR, 47.5% had initial shockable rhythm, and 7.6% were delivered shock by AEDs), it was difficult to define the duration of cardiac arrest and these data are lacking in our logistic regression model. Therefore, further study is needed to confirm our results using more detailed prehospital data. Third, as mentioned above, there are several Japanese specific EMS system and strategy of termination procedure related limitations that can affect the outcome of the patients; therefore, the generalisability should be interpreted with caution. Fourth, patients with fever prevention strategy are lacking in our analysis, because TTM was performed according to the former guidelines [37, 38], although more recent trials have selected this strategy [11, 12]. Therefore, further study is needed to clarify the effect of the cooling method at higher target temperatures. Finally, the long-term outcome showed no significant difference between SC and EC, although this was not our primary outcome, and the available data was limited (Additional file 2: Table S2). Because the outcome of OHCA patients may be modified after 1 month [39], further study should also focus on this issue.

## Conclusions

From the nationwide OHCA registry in Japan, we suggested that the use of EC was associated with better outcomes in OHCA patients with an initial non-shockable rhythm, while no such association was observed in those with initial shockable rhythm.

## Supplementary Information

**Additional file 1: Table S1.** Standardised mean difference of baseline characteristics of the study population before and after adjustment of IPW according to the cooling method.

**Additional file 2: Table S2.** Long-term outcome of the study population according to the cooling method.

## Data Availability

The data that support the findings of this study are available from the JAAM–OHCA registry committee. Restrictions apply to the availability of these data, which were used under license for the current study, and so are not publicly available. However, data are available from the authors upon reasonable request and with permission from the JAAM–OHCA registry committee.

## Abbreviations

TTM: Target temperature management
OHCA: Out-of-hospital cardiac arrest
SC: Surface cooling
EC: Endovascular cooling
JAAM: Japanese Association for Acute Medicine
ECMO: Extracorporeal membrane oxygenation
CPR: Cardiopulmonary resuscitation
AED: Automated external defibrillator
EMS: Emergency medical service
PCI: Percutaneous coronary intervention
CPC: Cerebral performance category
OR: Odds ratio
AOR: Adjusted odds ratio
CI: Confidence interval
IPW: Inverse probability weighting
